# Primary Care Physicians' Experience with Electronic Medical Records: Barriers to Implementation in a Fee-for-Service Environment

**DOI:** 10.1155/2009/853524

**Published:** 2008-12-04

**Authors:** D. A. Ludwick, John Doucette

**Affiliations:** ^1^Department of Mechanical Engineering, Faculty of Engineering, University of Alberta, Edmonton, Alberta, Canada T6G 2G8; ^2^Sherwood Park - Strathcona County Primary Care Network, Sherwood Park, Alberta, Canada T8A 4W6; ^3^TRLabs, #401, 9426, 51 Avenue NW, Edmonton, Alberta, Canada T6E 5A6

## Abstract

Our aging population has exacerbated strong and divergent trends between health human resource supply and demand. One way to mitigate future inequities is through the adoption of health information technology (HIT). Our previous research showed a number of risks and mitigating factors which affected HIT implementation success. We confirmed these findings through semistructured interviews with nine Alberta clinics. Sociotechnical factors significantly affected physicians' implementation success. Physicians reported that the time constraints limited their willingness to investigate, procure, and implement an EMR. The combination of antiquated exam room design, complex HIT user interfaces, insufficient physician computer skills, and the urgency in patient encounters precipitated by a fee-for-service remuneration model and long waitlists compromised the quantity, if not the quality, of the information exchange. Alternative remuneration and access to services plans might be considered to drive prudent behavior during physician office system implementation.

## 1. Introduction

Aging populations with complex health conditions such as obesity and chronic disease place an increasing burden on primary care systems in many countries [1–5]. While demand escalates, health human resource supply is declining as Canada's health workforce retires earlier and the average age of the remaining working population increases [6–8]. Higher training requirements, tuition fees [9], certification requirements [8, 10] and a higher female to male enrolment ratio are leading to a decline in the primary care physician workforce. The adoption of health information technology (HIT) is seen as one way to address the widening health care demand and supply gap [11–13]. It seems intuitive that HIT would improve patient safety, improve physician office efficiency and mitigate shortages in health human resources, but studies have shown that such systems can compromise short-term physician office performance [14–16], intimidate physicians and their office staff [17], and have shown, on occasion, to increase medical errors [18–20].

Health information system adopters face several risks when implementing health systems [21]. The purpose of this project was to assess the relevance and impact of these risks in the context of primary care in Sherwood Park, Alberta. Due to an economic boom, the population of Sherwood Park grew 14% from 2001 to 2006 compared to a population growth rate of 10% in Edmonton [22] (the nearest city) and 5.4% in Canada [23] during the same period. The Sherwood Park circumstance offers a microcosm in which to study the effects of HIT adoption in primary care.

Primary care usually refers to family or general practice and is the first point of contact a person has with the health system [24]. An *electronic medical record* is a computerized health information system where providers record detailed encounter information such as patient demographics, encounter summaries, medical history, allergies, intolerances, and lab test histories. Some EMRs support scheduling, billing, reporting, order entry, results management, and decision support [25–27]. Such systems are often referred to as *physician office systems* or *practice management systems* [25, 28].

## 2. Previous Research

In a previous comprehensive literature review [21], the implications of HIT were examined across a number of care domains. Health information technology implementation success depends on a number of factors. Implementers need to be aware of sociotechnical system fit to achieve success [29–32]. However, implementers perceive privacy [33, 34], patient safety, provider/patient relations, staff anxiety [31], time needed to implement [35–39], quality of care, financial [40–42], efficiency, and liability [43] factors as risks that can pressure or derail a project. Users' previous experiences with HIT affected their experience with a new system, both positively and negatively [44–46]. Users applied their previous experience to new systems and evaluated the usability and effectiveness of their new system against that of the previous system. Exam room layouts and computer monitor placement have been shown to affect, positively and negatively, the interaction between provider and patient [47–49]. Implementers can insulate the project from such risks by establishing strong leadership [16, 37, 45, 50–54], using project management techniques [50, 51, 55–61], establishing standards, and training their staff [13, 16, 35–39, 46, 52, 54, 58, 62–65] to ensure such risks do not compromise implementation success.

## 3. Methodology

This research project used one hour semistructured interviews to acquire information from primary care physicians' experience of selecting, implementing, and operating an EMR system. Physician candidates were selected from our local primary care network, in which 47 physicians are members. Inclusion criteria required physicians to be practicing full time in the community, have significant EMR experience and be a lead physician or influencer in clinic decision making. Physicians were paid a honorarium to acknowledge their lost revenue generating opportunity. An interview guide consisting of closed-ended statistical questions and several open-ended questions stimulated a qualitative conversation regarding the experience. The researcher recorded detailed notes that were later used for synthesis and analysis.

**Figure 1:**
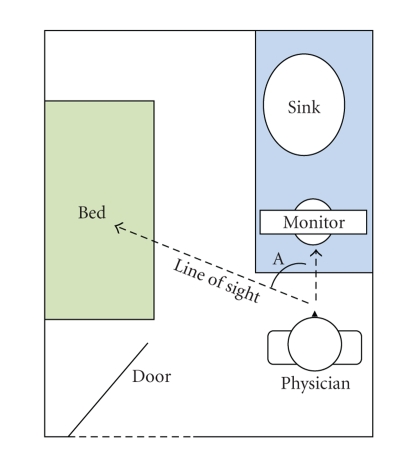
Exam room layout 1.

**Figure 2:**
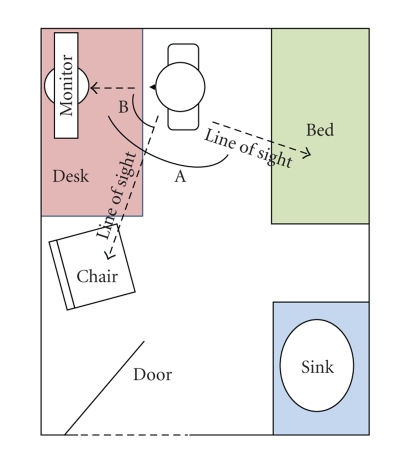
Exam room layout 2.

**Figure 3:**
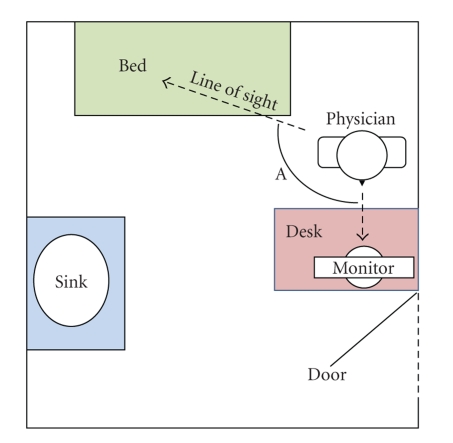
Exam room layout 3.

After the interview, the researcher documented the layout of exam rooms. Exam rooms were depicted in a floor plan conceptually locating the computer keyboard and monitor with respect to the patient exam table or chair. The researcher also asked physicians to recount the positioning of the patient with respect to themselves and the computer. Exam room layouts were subsequently analyzed and categorized into three general types for critical review. Figures 1, 2, and 3 depict the three exam room layouts which best illustrate the wide range of layouts. The researcher recorded the quantity of rooms in the clinics for statistical purposes.

## 4. Results

Of the 47 physicians in the primary care network, 19 physicians are clinic leads. Of the 19 clinic owners or influencers, there are 11 clinics with practical EMR experience. Nine interviewees were selected who represent a total of 26 physicians and were interviewed during the months of February and March 2008 using the interview guide shown in Table 1. Two interviewees were sole practitioners, 2 interviewees represented clinics with 2 physicians each in them, 3 interviewees represented practices with of 3 physicians, one interviewee represented a clinic of 5 physicians, and one interviewee represented a clinic of 6 physicians. Table 2 summarizes the key findings from the interviews' closed-ended questions.

All physicians have at least 10 years of practice experience. Two physicians were female, both of which are operating in multiphysician practices. All interviewees except one considered themselves owners or decision makers in the practice but all reported that they had a hand in selecting and implementing their EMR. Eight physicians are satisfied with their own computer and data entry skills, rating themselves a 3 out of 5 or higher. Physicians have 30 patient encounters per day but, often see as many as 40 patients at roughly 10 minutes per encounter.

Seven physicians routinely make encounter notes directly into their EMR during the interview, although occasionally they complete note taking outside the room after the encounter. The other 2 physicians make notes on paper. Six physicians have permanently stationed desktop computers located in exam rooms to make notes, while two use wireless laptop computers. One of the two physicians using a paper system has computers stationed in his exam rooms but has reverted back to record patient encounter data on paper. One clinic reported that physicians wrote encounter notes on paper and scanned them into their EMR as a way to kick start the implementation and develop their computer skills. Eight clinics use paper record systems prior to their EMR, while one clinic is now operating its second EMR.

Eight physicians did not follow a prescribed procurement plan while the other followed a procurement plan consisting of a market scan, price analysis, vendor demonstrations, and visiting colleagues' clinics. Four physicians invited vendors to demonstrate their products to them at their clinics. Two physicians completed a price comparison, while one called their professional association for procurement advice, another acquired his EMR through personal connections, and yet another could not remember how he had selected his EMR. Physicians did not have the time or experience to follow a detailed procurement plan. All physicians reported disorientation in the procurement process as they had not had any related experiences in the past.

Physicians did not report the breakpoint that McGrath had reported [47]. Even though physicians said patients rarely commented, some physicians felt a need to apologize for taking notes on computer, or at least to acknowledge it to patients. Those physicians who had owned their system for a while were more comfortable since most patients had rotated through and seen the system previously. Physicians felt compelled to stop typing if patients became emotional during the interview, although they did not always do so.

Our physicians complained about their training and postsale experience with their vendor. Instead of a training regimen similar to that described in the literature [21], physicians reported that their vendor simply offered one training session of one half to a full day in duration. Training was often too soon after implementation. Physicians had not developed sufficient experience with their new EMR to ask relevant questions or appreciate the answers.

Physicians reported that they could not always access vendor technical support. Even when they could get a person, they were not confident that the technical support person “knew how a clinical practice functioned.” Physicians were concerned that the company did not appreciate the implications of a dysfunctional EMR. Physicians often procured supplementary local technical support at higher cost.

Physicians pointed to opportunities for more efficient data entry. Two physicians have made great use of the template features in their EMRs. They have spent significant time building templates which allow them to enter data or orders into their system for common ailments with a few key strokes. Two other physicians reported that they have made use of voice recognition software which emulates dictation, a familiar mode of data entry for physicians. Voice recognition software requires training and is not functional for clinicians with strong accents, but physicians who invested significant time training their software had achieved a satisfactory level of efficiency.

A total number of 19 examination rooms were viewed during the study representing 51% of the total 37 rooms in these physicians' offices. Figures 1 to 3 depict three exam room layouts which were categorized based on the following observations:

- the presence of an office desk, or not,
- the presence of a patient interview chair, or not,
- the general size of the room,
- the orientation of the computer monitor with respect to the physician, the exam table, and the patient,

Table 3 below summarizes the key characteristics of the observed exam rooms. The layout-type column indicates the figure which best depicts the exam room. Eight out of 9 physicians interview patients while they are seated in chairs (one owns rooms numbered 13 and 14 which were too small for a chair and therefore interviewed and examined the patient on bed). Note that two columns in the table refer to an angle. Exam room observations note that an angle was created between the lines of sight from physician to monitor and physician to bed (Angle A) as well as between the lines of sight from physician to monitor and physician to chair (Angle B), if the chair existed.

Many brands of EMR are used in these clinics (Telin, Global Biometrics, Med Access, Practice Solutions, EMIS, and Wolf). Clinics use most system features including billing, scheduling, importing lab results, drug order entry, and encounter note taking. Drug-to-drug and drug-to-allergy contraindication management was used by many physicians when the data had been entered to support it. Three physicians do not use contraindication management because they leave this responsibility to the pharmacy. One practice reported that this feature had to be purchased separately so was not currently part of their system. Many physicians automatically receive lab test results electronically through an electronic mailbox system (ftp-based system) arranged by the RHA.

## 5. Discussion

The purpose of this project was to assess the relevance and impact of risk and insulating factors for HIT adoption in the context of primary care in Sherwood Park, Alberta. Our interviews showed that few physicians follow a complete procurement approach. Exam room layouts require computer systems to be situated such that physicians face away from their patients. Physicians struggle to get appropriate training and technical support for their systems. However, when physicians invest the time, they realize benefits to using their EMR.

Time constrains many physician offices when procuring and implementing HIT. In Canada, primary care physicians get paid on a fee-for-service basis. The more patients they see, the more revenue they generate. Further, Canada reports large wait times for access to health services [66]. Physicians choose not to invest the time in systems procurement because they are uncomfortable with the process. Investigating systems during office hours reduces revenue generating opportunity and increases patient wait times. Interestingly, other reviews have shown that pay-for-performance models, as one strategy for payment, have worked well in driving to long-term national HIT adoption success [67]. More research on the effects of remuneration models on adoption is warranted and will be the subject of future research.

Interviews revealed that exam room layouts could compromise the quantity, if not quality, of information transfer from patient to physician. Our figures above attempt to simplify and categorize these into three types based on the type and placement of furniture, the type and placement of the computer monitor, as well as the positioning of the physician with respect to the patient. If the amount of interpersonal communication is a function of visual cues, as would be suggested by Mehrabian [68], then the Angle A, created between the two lines of sight from the physician to their computer monitor and the physician to the patient, would be critical to the success of communications. Layout 1 has a relatively small angle (estimated at 60 degrees). Layouts 2 and 3 show Angle A to be greater than 90 degrees. This situates the patients somewhat behind the physician as they face the monitor. Physicians operating in exam rooms similar to that of layout 1 expressed the least concern over eroded interpersonal communications. Furthermore, the two physicians using laptops could position themselves to look over their laptop monitor directly at the patient, effectively reducing Angle A to zero degrees. We did not interview enough physicians to be conclusive, but we assert that there could be a relationship between the quantity, and possibly quality, of information transfer from patient to physician and the size of Angle A, as would be supported by Robinson et al. [69]. The smaller Angle A is, the more direct patient eye contact is and, therefore, the more complete the interpersonal communication, possibly leading to higher quality of care. A few physicians appreciated this concept as one had previously taken advantage of pending renovations to accommodate her systems implementations and another was planning changes to his office furniture to close Angle A to zero degrees.

The above problem gets more aggravated when we consider our physicians' computer skills in the context of the complex EMR user interfaces and the time pressure of a patient encounter within the context of a fee-for-service remuneration model. Our physicians self-reported their computer skills rated at 3, on a scale of 1 to 5. Similarly, a US survey [70] reported that their physician survey respondents felt quite confident about their computer skills. We did not observe physicians using their EMR for note taking during patient encounters (exam room observation would have required significant ethics approval); however, extrapolating complaints they had about the usability of basic computer functions make us hypothesize that physicians, vendors, and HIT advocates have underestimated the level of computer skills required for this work (physicians reported that they hunt for menus and buttons to the extent they sometimes stop using the EMR in interviews because of the disruption). EMR user interfaces are complex and busy (reminiscent of an airplane cockpit). The skills needed to listen to patients' complaints, assess medical relevance, contemplate interventions as well as type notes—all at the same time–would require a significant level of concentration, typing skills, and familiarity with the application's user interface, not normally found in the most adept computer users. Therefore, we were not surprised to learn that physicians often had to complete note taking after the encounter or at the end of the business day. We hypothesize that HIT can disrupt the flow of information from patient to provider when computer monitors require the physician to face away from the patient. Physicians' eyes are focused on the computer system and not the patient which compromises information transfer especially in clinics with high-patient volumes and inexperienced physician computer users. We are concerned that this may compromise the physicians' implied and historic role as confidante. We are planning future research to investigate this concept further.

The study's most obvious weakness is its narrow field of interviewees. Sherwood Park PCN has over 40 physicians; however, only nine met our inclusion criteria. The small sample means that the discussion and conclusions outlined above can only be considered directional. They are not conclusive or statistically significant. Bias may result from interviewee selection. Ideally, interviewees would have represented more clinics from a greater geographical area. We interviewed physician leaders who influenced implementation decisions; yet, physician leaders' perceptions may not reflect those of their associates. Our physicians are members of a PCN; consequently, findings may not be applicable to primary care physicians who practice outside of an interdisciplinary team. We infer that there is a relationship between information transfer and the angles described above. Future research involving patients is required to confirm this. This Alberta study is influenced by provincial matters, such as health policy, remuneration approaches, and physician office system funding models, which may prevent results from applying in other jurisdictions.

## 6. Conclusions

Our interviews and previous research have shown time to be a precious resource for physicians in several facets of their day-to-day operations. Physicians do not take the time to properly become familiar with the available products, select an EMR, implement it, and then train to use it even though colleagues have invested time and realized great benefit. We wonder whether the current fee-for-service payment model in Alberta creates an urgency to maintain patient throughput. The opportunity to maximize clinic revenue and waiting rooms full of patients may discourage physicians from investing the time in EMR implementation activities. The Sherwood Park experience might point to a need for a change in remuneration approach and guidance for reducing wait times, at least for the purposes of selecting, acquiring, and implementing the system prior to returning to steady-state clinic operations.

Computer skills, complexity in EMR interfaces, and exam room layouts combine to affect physicians' encounter experience. Despite their strong self assessments, we are concerned that physicians do not have sufficient computer skills to take notes and navigate an EMR while listening to a patient in an encounter. Physicians might consider changing to laptop systems (even with wired networks), using voice recognition software and/or developing templates to permit more direct patient interaction and improve efficiency.

## 7. Relevance

Alberta, like other jurisdictions, is aggressively driving the adoption of HIT. Despite well structured and financed programs, factors such as computer aptitude in physicians and complexity in graphical user interfaces are not being considered as hindrances to adoption. Medical associations provide valuable coaching to physicians on system procurement and physician office design, but time constrains physicians from taking advantage. Vendor certification programs test and conform EMR applications for interoperability but need to increase scrutiny on vendor business and technical support qualifications. Although jurisdictions continue to finance adoption, organized effort needs to be applied to other points of friction. Training for physicians on computers, establishing user interface design standards and guidance on exam room design is also required.

Canada's fee-for-service payment model provides physicians with an opportunity to maximize patient throughput. Yet, HIT projects take physicians offline from their core activities as physicians. When physicians are remunerated based on patient volume, they are discouraged from spending the time needed to make their implementations a success. This paper does not advocate one payment model over another, but simply points to a pattern of behavior which seems to be caused by the current approach. Jurisdictions might consider the implications of the current payment model with regard to adoption and provide alternative vehicles which encourage physicians to invest the time to maximize outcomes from their investments.

## Figures and Tables

**Table 1:** Interview guide.

Interview questions for physician interviews		
Sherwood Park - Strathcona County PCN		
Interviewee(s):		
Interviewer:		
Date:		

Interview questions	Notes of candidate's answer	Interviewer's guide to answers
How long have you been in practice?		[Years or Months]
How many physicians are currently practicing in your office?		[Number]
How many non-physician clinicians do you employ?		[Number]
How many staff/admin do you employ?		[Number]
Are you the practice owner/key decision maker? If not, what is your role?		[Yes/No]. [If no, partner, contracted, part time]
How many patients do you typically see in a day?		[Number]
What is your target interview duration?		[Minutes]
What sort of health records system do you currently use?		[paper; electronic, but paper used to record notes first followed by transcription; electronic, desktop in exam room; electronic, laptop carried into exam room]
Can you describe the role your health information system plays when you are interviewing a patient		[take paper based notes as I go, take e-based notes as I go, don’t take any notes in interview]
How long have you owned your EMR?		[Years or Months]
On a scale of 1 to 5, where 1 is poor and 5 is excellent, can you rate your computer skills (before and after the implementation)?		[1 to 5], [1 to 5]
When/where do you make your encounter notes?		[during interview in exam room, immediately after interview outside exam room door, at end of day either at the office or at home]
Prior to your current practice, what did you use for health information system to support your work?		[paper; electronic, but paper used to record notes first followed by transcription; electronic, desktop in exam room; electronic, laptop carried into exam room]
Can you describe the process you went through to buy your EMR? How did you gather market information?		[market scan, called vendors directly, talked to colleagues, talked to AMA/POSP/CPSA]
How did you select your EMR? What purchasing factors were most relevant to you?		[price, features, eligibility for financial support]
How did you install the EMR into your practice?		[big-bang, pilot, team-oriented integrative approach]
What do you use your EMR system for?		[Billing, scheduling, encounter note taking, lab results, order entry, contraindication management]
Where do you get your technical support?		[self, colleague, 3rd party]
What do you like/dislike about your current system?		—
Did you notice a change in your patient volumes after your implementation? If so, can you say what % age it dropped to and for how long? Why?		[%, months]
On a scale of 1 to 5, where 1 is completely dissatisfied and 5 is extremely satisfied, what would you say your overall satisfaction is with your system?		[1 to 5]
Knowing what you know now, would you still have bought the EMR? Why do you say that?		[yes/no]

**Table 2:** Closed-ended interview results: statistics describing the number and experience of physicians, patient throughput, years of experience using an EMR, computer skills, and clinic size.

Interview factor	Average (*N* = 9)	Range (*N* = 9)
Years in practice	20 years	10 to 33 years
Number of physicians practicing in clinic	3	1 to 6
Number of nonphysician clinicians in practice	1.75	0 to 8
Number of support staff in clinic	2.5	1 to 4
Target number of patients to be seen in a day	32.5	20 to 40
Target patient interview duration	8.4 minutes	7.5 to 15 minutes
Number of years owned an EMR	4 years	0 to 10 years
Personal rating of computer skills (range: 1 to 5)	3.25	2 to 4
Overall EMR satisfaction rating (range: 1 to 5)	2.9	2 to 4.5

**Table 3:** Exam room layout results.

Room no.	Layout type	System configuration	Has chair	Has office desk	Room size	Angle A (monitor to bed)	Angle B (monitor to chair)
1	2	Desktop	Yes	Yes	Medium	120	90
2	2	Desktop	Yes	Yes	Medium	120	90
3	2	Desktop	Yes	Yes	Medium	120	90
4	3	Desktop	No	Yes	Large	120	n/a
5	2	Desktop	Yes	Yes	Large	120	120
6	1	Laptop	Yes	No	Medium	0	0
7	1	Laptop	Yes	No	Medium	0	0
8	1	Laptop	Yes	No	Medium	0	0
9	2	Desktop	Yes	Yes	Small	180	90
10	2	Desktop	Yes	Yes	Small	180	90
11	3	Desktop	Yes	Yes	Large	180	180
12	2	Desktop	Yes	Yes	Large	120	90
13	1	Desktop	No	Yes	Small	90	n/a
14	1	Desktop	No	Yes	Small	90	n/a
15	2	Desktop	Yes	Yes	Medium	180	90
16	2	Desktop	Yes	Yes	Medium	180	90
17	1	Laptop	Yes	No	Medium	0	0
18	1	Laptop	Yes	No	Medium	0	0
19	1	Laptop	Yes	No	Medium	0	0
